# Further study of circulating IgG antibodies to CD25‐derived peptide antigens in nonsmall cell lung cancer

**DOI:** 10.1002/2211-5463.12034

**Published:** 2016-02-16

**Authors:** Cairen Chen, Weili Wang, Qingyong Meng, Ning Wu, Jun Wei

**Affiliations:** ^1^ School of Clinical Laboratory ScienceGuangdong Medical UniversityDongguanChina; ^2^ Department of Radiation OncologyAugusta UniversityGAUSA; ^3^ Department of Radiation OncologyChina‐Japan Union HospitalJilin UniversityChangchunChina; ^4^ Division of Health ResearchUniversity of the Highlands & IslandsCentre for Health ScienceInvernessUK

**Keywords:** antibody test, CD25, nonsmall cell lung cancer, tumor immunity

## Abstract

A recent study reported that circulating antibodies to CD25‐derived peptide antigens were significantly higher in patients with nonsmall cell lung cancer (NSCLC) than control subjects. The present study was, thus, undertaken to replicate the initial finding with different sample sets. An in‐house ELISA was applied to determine circulating IgG antibodies to linear peptide antigens derived from CD25. A total of 111 patients with NSCLC and 216 control subjects were recruited and divided into the discovery sample (51 vs 108) and the validation sample (60 vs 108) based on the time of sampling. Student's *t* test showed that circulating anti‐CD25 IgG levels were significantly higher in the patient group than the control group (*t* = 2.23, *P* = 0.027) and the validation sample replicated this finding (*t* = 3.31, *P* = 0.0012), generating a combined *P* value of 0.0004 (χ^2^ = 20.8, *df* = 4). Fisher's combining probability revealed that patients with stage IV NSCLC had a significant increase in anti‐CD25 IgG levels compared with control subjects (χ^2^ = 22.1, *df* = 4, *P* = 0.0002) but those with the other three stages did not. This study suggests that circulating anti‐CD25 IgG antibodies may have prognostic rather than early diagnostic values for lung cancer.

**Abbreviations**
CD25: alpha chain of interleukin 2 (IL‐2) receptors
ELISA: enzyme‐linked immunosorbent assay
FOXP3: forkhead/winged helix transcription factor
IgG: immunoglobulin G
IL‐2: interleukin 2
NC: negative control
NSCLC: nonsmall cell lung cancer
OD: optical density
SBI: specific binding index
TAAs: tumor‐associated antigens
Treg: regulatory T

Regulatory T (Treg) lymphocytes are a subset of CD4^+^ T cells and play a role in maintaining immunologic homeostasis and peripheral tolerance to self‐ and allo‐antigens 1, 2. The majority of Treg cells can be identified phenotypically by the expression of the alpha chain of interleukin 2 (IL‐2) receptors, also known as CD25 3, and forkhead/winged helix transcription factor (FOXP3). Treg cells suppress T‐cell immune responses by the inhibition of antigen‐presenting cells such as dendritic cells 4 or by the secretion of some cytokines like IL‐10, TGF‐β, IL‐27 and IL‐35 5, 6. Thus, Treg cells are likely to be involved in the escape of cancer cells from antitumor effector T cells 7. Increased numbers of Treg cells have been in patients with different types of cancers 8, 9, 10, 11, 12. These observations raise the possibility that a compensatory response to the increased density of Treg cells can be triggered in the body. If this was a case, autoantibodies against CD25 may be altered in order to down‐regulate the function of Treg cells in malignant diseases.

In recent studies, circulating antibodies to CD25 have been found to be increased in patients with solid cancers, including lung cancer 13, esophageal cancer 14 and breast cancer 15. All these studies applied an in‐house ELISA, which was developed with CD25‐derived linear peptide antigens, to detect circulating anti‐CD25 antibodies. To replicate this initial finding, the present study was undertaken to detect anti‐CD25 IgG levels in patients with lung cancer and control subjects from different sample collections, including the discovery sample and the validation sample.

## Methods

### Subjects

A total of 111 patients who were newly diagnosed as having nonsmall cell lung cancer (NSCLC) were recruited by the Fourth Affiliated Hospital of China Medical University, Shenyang 110032, China, in the period between March 2013 and November 2014. Of these 111 patients aged 64.0 ± 10.4 years, 70 were male and 41 were female; they were divided into the discovery samples (*n* = 51) that were collected during 2013 and the validation samples (*n* = 60) that were collected during 2014. Their diagnosis and tumor staging were made based on radiographic examination and histological confirmation; inclusion of patients was restricted to those with adenocarcinoma and squamous carcinoma only. Blood samples were taken prior to any anticancer treatment. Over the same time period, a total of 216 healthy subjects, well matched in age (59.1 ± 3.5 years) and smoking history, were also recruited from local communities, 108 of whom were used as controls for the discovery samples and 108 for the validation samples. All the samples from Clinical interview and radiographic examination were applied to rule out the control subjects who had history of lung cancer or any other malignant tumors. All the subjects were of Chinese Han origin and they all gave informed written consent to participate in this study as approved by the Ethics Committee of the Fourth Affiliated Hospital of China Medical University, and conformed to the requirements of the Declaration of Helsinki.

### Autoantibody testing

Circulating anti‐CD25 IgG was detected with a modified ELISA method that was developed using a linear peptide antigen derived from human CD25 protein according to previous studies 13, 14, 15, 16, 17; a control antigen was designed based on a maize protein (NCBI accession 1BFA_A), and its sequence is as follows:

H‐ H‐HAQLEGRLHDLPGCPREVQRGFAATLVTN‐OH. In brief, both the CD25‐derived peptide antigen and control antigen were synthesized by solid‐phase chemistry with a purity of > 95%, and then applied to develop a relative ELISA test for the detection of circulating IgG antibodies to CD25. The synthetic peptides were dissolved in 67% acetic acid to obtain a concentration of 5 mg·mL^−1^ as stocking solution kept at −20 °C, and were diluted with phosphate‐buffered saline (PBS)‐based coating buffer (P4417, Sigma‐Aldrich) containing 0.1% sodium azide. Coaster 96‐Well Microtiter EIA Plates (ImmunoChemistry Technologies, USA) were half‐coated in 0.1 mL·well^−1^ of CD25‐derived antigens and half‐coated in 0.1 mL·well^−1^ of the control antigen. The antigen‐coated 96‐well microplate was covered and incubated overnight at 4 °C. After the antigen‐coated plate was washed three times with the wash buffer made from Tris‐buffered saline with Tween^®^20 (T9039, Sigma‐Aldrich), 100 μL plasma sample diluted 1 : 150 in assay buffer (PBS containing 1.5% BSA) was added and 100 μL assay buffer was also added to the negative control (NC) wells. Following 3 h incubation at room temperature, the plate was washed three times and 100 μL peroxidase‐conjugated goat antibody to human IgG (A8667, Sigma‐Aldrich) diluted 1 : 30000 in assay buffer was added to each well. After incubation at room temperature for an hour, color development was initiated by adding 100 μL Stabilized Chromogen (SB02, Life Technologies) and terminated 25 min later by adding 50 μL Stop Solution (SS04, Life Technologies). The measurement of optical density (OD) was completed on a microplate reader within 10 min at 450 nm with a reference wavelength of 620 nm.

Each sample was tested in duplicate. To reduce the interference from a nonspecific signal produced by passive absorption of various IgG antibodies in plasma to the surface of 96‐well microplate, a specific binding index (SBI) was used to express the levels of circulating anti‐CD25 IgG antibodies. SBI was calculated as follows:

$$\mathrm{SBI}=\left({\text{OD}}_{\mathrm{CD}25}-{\text{OD}}_{\mathrm{NC}}\right)/\left({\text{OD}}_{\mathrm{control}}-{\text{OD}}_{\mathrm{NC}}\right)$$

To minimize an intra‐assay deviation, the ratio of the difference between duplicated OD values to their sum was used to assess the precision for assay of each sample. If the ratio was found to be > 15%, the test of this sample was treated as being invalid and was not used for data analysis.

### Data analysis

Microsoft Excel 2010 was used to construct a database with individual SBI values. Student's *t* test was applied to examine the difference in SBI between the patient group and the control group; Fisher's combining probabilities were applied to work out the combined *P* values based on the *t* test performed on the discovery sample and the validation sample 18.

## Results

As shown in Table 1, the Student's *t* test showed that circulating anti‐CD25 IgG levels were significantly higher in patients with NSCLC than control subjects (*t* = 2.23, *df* = 157, *P =* 0.027) in the discovery sample, and the validation sample also replicated this alteration (*t* = 3.31, *df* = 166, *P =* 0.0012), generating a combined *P* value of 0.0004 (χ^2^ = 20.7, *df* = 4).

**Table 1:** Analysis of circulating levels of IgG antibodies to CD25 in NSCLC

Sample	Patient (*n*)	Control (*n*)	*t* a	*P* b
Discovery	0.99 ± 0.20 (51)	0.92 ± 0.17 (108)	2.23	0.027
Validation	0.85 ± 0.18 (60)	0.77 ± 0.13 (108)	3.31	0.0012

The antibody levels are expressed as mean ± SD in SBI.

a Student's *t* test (two‐tailed).

b Combining probabilities: χ^2^ = 20.7, *df* = 4, *P* = 0.0004 for anti‐CD25 IgG levels.

Further analysis was performed to clarify if the anti‐CD25 IgG levels were significantly increased in all four stages (I, II, III, and IV) of NSCLC. The results showed that patients with stage IV NSCLC had a significant higher levels of circulating anti‐CD25 IgG than control subjects in both the discovery sample (*t* = 3.10, *df* = 118, *P =* 0.002) and the validation sample (*t* = 2.71, *df* = 127, *P =* 0.008), generating a combined *P* value of 0.0002 (χ^2^ = 22.1 *df* = 4); however, patients with the other three stages of NSCLC failed to show a significant increase in circulating antibody levels (Tables 2 and 3).

**Table 2:** The levels of circulating anti‐CD25 IgG in different stages of NSCLC in the discovery sample

Stage	Patient (*n*)	Control (*n*)	*t* a	*P*
I	0.94 ± 0.15 (11)	0.92 ± 0.17 (108)	0.29	0.771
II	1.04 ± 0.40 (6)	0.92 ± 0.17 (108)	1.52	0.494
III	0.95 ± 0.11 (22)	0.92 ± 0.17 (108)	0.73	0.468
IV	1.09 ± 0.23 (12)	0.92 ± 0.17 (108)	3.10	0.002

The antibody levels are expressed as mean ± SD in SBI.

a Student's *t* test (two‐tailed).

**Table 3:** The levels of circulating anti‐CD25 IgG in different stages of NSCLC in the validation sample

Stage	Patient (*n*)	Control (*n*)	*t* a	*P* b
I	0.84 ± 0.18 (13)	0.77 ± 0.13 (108)	1.90	0.06
II	0.88 ± 0.16 (7)	0.77 ± 0.13 (108)	2.17	0.032
III	0.82 ± 0.16 (19)	0.77 ± 0.13 (108)	1.62	0.107
IV	0.86 ± 0.20 (21)	0.77 ± 0.13 (108)	2.71	0.008

The antibody levels are expressed as mean ± SD in SBI.

a Student's *t* test (two‐tailed).

b Combining probabilities calculated based on the *P* values from the two samples (Tables 2 and 3) in the 4 stages of NSCLC: χ^2^ = 6.2, *df* = 4, *P* = 0.185 for stage I, χ^2^ = 8.3, *df* = 4, *P* = 0.082 for stage II, χ^2^ = 6, *df* = 4, *P* = 0.199 for stage III and χ^2^ = 22.1, *df* = 4, *P* = 0.0002 for stage IV.

## Discussion

This study has replicated the initial findings that circulating levels of anti‐CD25 IgG antibodies were significantly increased in NSCLC 13, although only stage IV NSCLC patients showed a significant increase in the anti‐CD25 IgG levels (Tables 2 and 3). This observation suggests that circulating anti‐CD25 IgG could serve as a biomarker for prognosis of NSCLC rather than early diagnosis of this malignancy.

Autoantibodies to tumor‐associated antigens (TAAs) have been extensively investigated for many years, but the biological significance of increased autoantibody levels in the circulation has yet to be clarified 19, 20, 21, 22, 23. While testing of autoantibodies to TAAs may have a potential benefit for early diagnosis of cancers, the association of these autoantibodies with prognosis of cancers has been shown to be inconsistent across studies. For an example, the autoantibody to tumor‐suppressor antigen p53 was reported to be associated with decreased survival rates in ovarian cancer by a couple of studies 24, 25 but with an increased survival rate by others 26, 27. The utility of circulating antibodies to TAAs as prognostic biomarkers may need further confirmation in large‐scale clinical studies. Based on our present results, anti‐CD25 IgG may have prognostic values for lung cancer, but further investigation is needed to replicate this initial work and to analyze the correlation between circulating anti‐CD25 IgG levels and the survival rate of patients with NSCLC.

There are a couple of limitations to this study. First, the sample size was rather small, and the study was underpowered to examine the significance for small effect size. Second, there was a big deviation in the IgG measurements between the discovery sample and the validation sample. Such a deviation may result from the duration of sample storage as well as system errors due to laboratory manipulation; however, both sample sets applied parallel control samples so that the experimental data should be reliable. Lastly, a significant increase in circulating IgG levels was observed only in patients with stage IV NSCLC, suggesting that circulating anti‐CD25 IgG could serve as a biomarker for prognosis of NSCLC rather than early diagnosis of the malignant disease. These initial findings still need to be prospectively validated in a larger cohort study.

## Conclusion

Circulating IgG antibodies to CD25 appear to serve as a biomarker for prognosis of nonsmall cell lung cancer rather than an early diagnostic value.

## Author contributions

QM and JW conceived of the study, recruited patients, and corrected the manuscript; CC and NW performed all laboratory work and drafted the manuscript; WW was mainly involved in supervising lab work, data analysis, and proofreading. All authors read the final version of the manuscript and approved the submission.
